# Supplementary material for: A proteogenomic analysis of clear cell renal cell carcinoma in a Chinese population
Source: Nat Commun. 2022 Apr 19;13:2052. doi: 10.1038/s41467-022-29577-x (PMC9019091; doi:10.1038/s41467-022-29577-x)
Supplement: Supplementary file 1 — Supplementary Information [file 41467_2022_29577_MOESM1_ESM.pdf]

Supplementary Information

A Proteogenomic Atlas of Clear Cell Renal Cell Carcinoma in a Chinese Population

Yuanyuan Qu, Jinwen Feng, Xiaohui Wu, Lin Bai, Wenhao Xu, Lingli Zhu, Yang Liu, Fujiang Xu, Xuan Zhang, Guojian Yang, Jiacheng Lv, Xiuping Chen, Guo-Hai Shi, Hong-Kai Wang, Da-Long Cao, Hang Xiang, Lingling Li, Subei Tan, Hua-Lei Gan, Meng-Hong Sun, Jiange Qiu, Hailiang Zhang, Jian-Yuan Zhao, Dingwei Ye, Chen Ding

Supplementary Figure 1

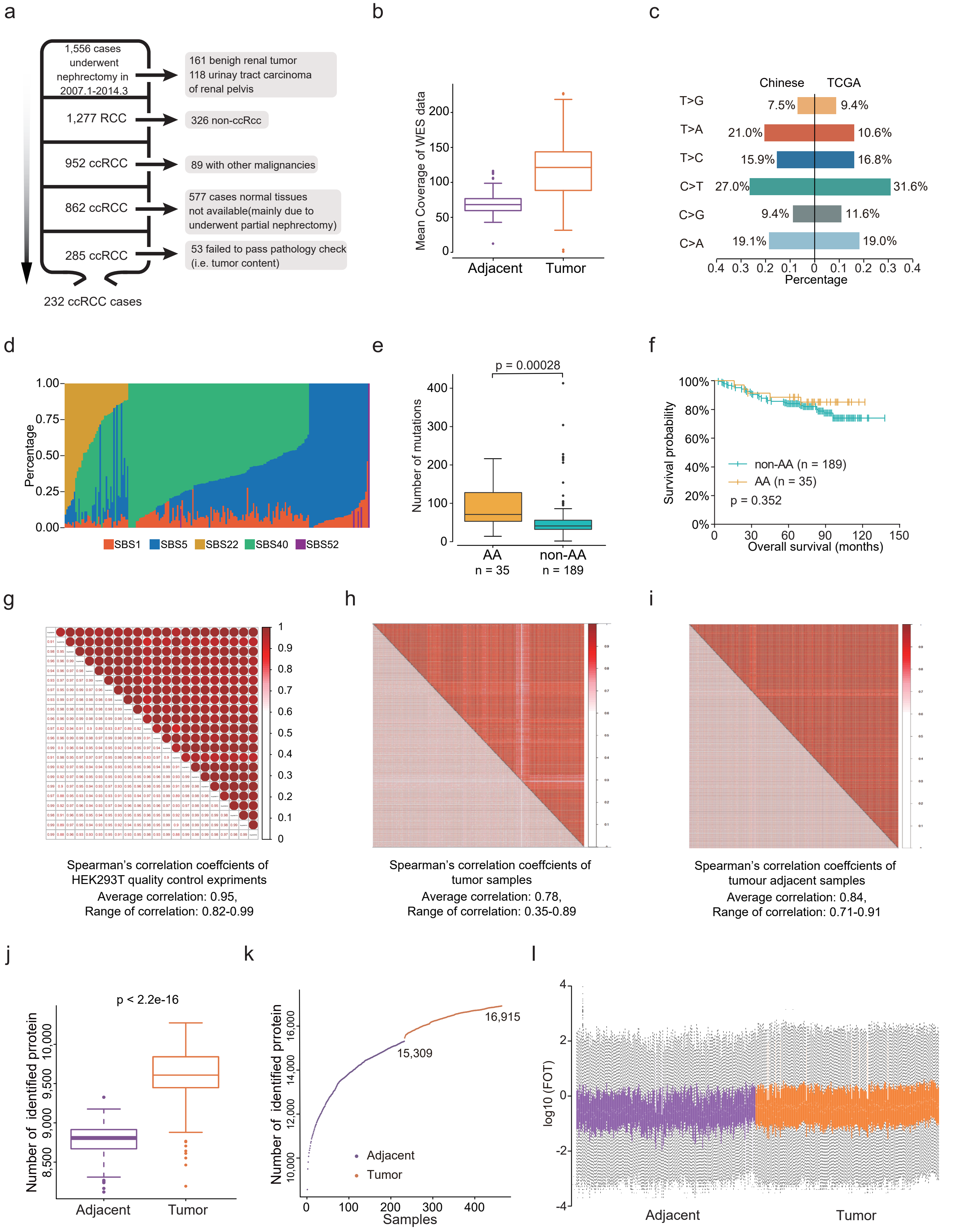

Supplementary Fig. 1 Sample Collection Criteria and Quality Assessments for WES and Proteome Data.

a, Sample collection process. **b**, Mean coverage of tumor and adjacent WES data (n = 224 pairs). **c**, Frequency of substitution mutations in the Chinese and TCGA cohorts. **d**, Relative percentage of each mutation signatures in 232 ccRCC cases. **e**, Boxplot of number of mutations in groups of patients with or without the AA signature. P value is derived from two-sided t test. **f**, Kaplan–Meier curves of OS for patients with or without AA signature (two-sided log-rank test). **g**, Longitudinal quality control of mass spectrometry using tryptic digests of HEK293T cells. The bottom panel represents the pairwise Spearman's correlation coefficients of the samples. **h**-**i**, Spearman's correlation coefficients of tumor-adjacent tissue and tumor tissue samples. The x and y axes represent log10-transformed protein abundances in each pairwise comparison. **j**, Box plots of proteins identified in tumors and adjacent tissues (n = 232 pairs). P value is derived from two-sided paired t test. **k**, Cumulative number of proteins identified as a function of sample numbers. **l**, Boxplot of the log10 (FOT) protein expression levels for the 232 pairs of tumor and adjacent tissue samples. Boxplots in panels **b**, **e**, **j**, l show the median (central line), the 25–75% IQR (box limits), the ±1.5×IQR (whiskers).

Supplementary Figure 2

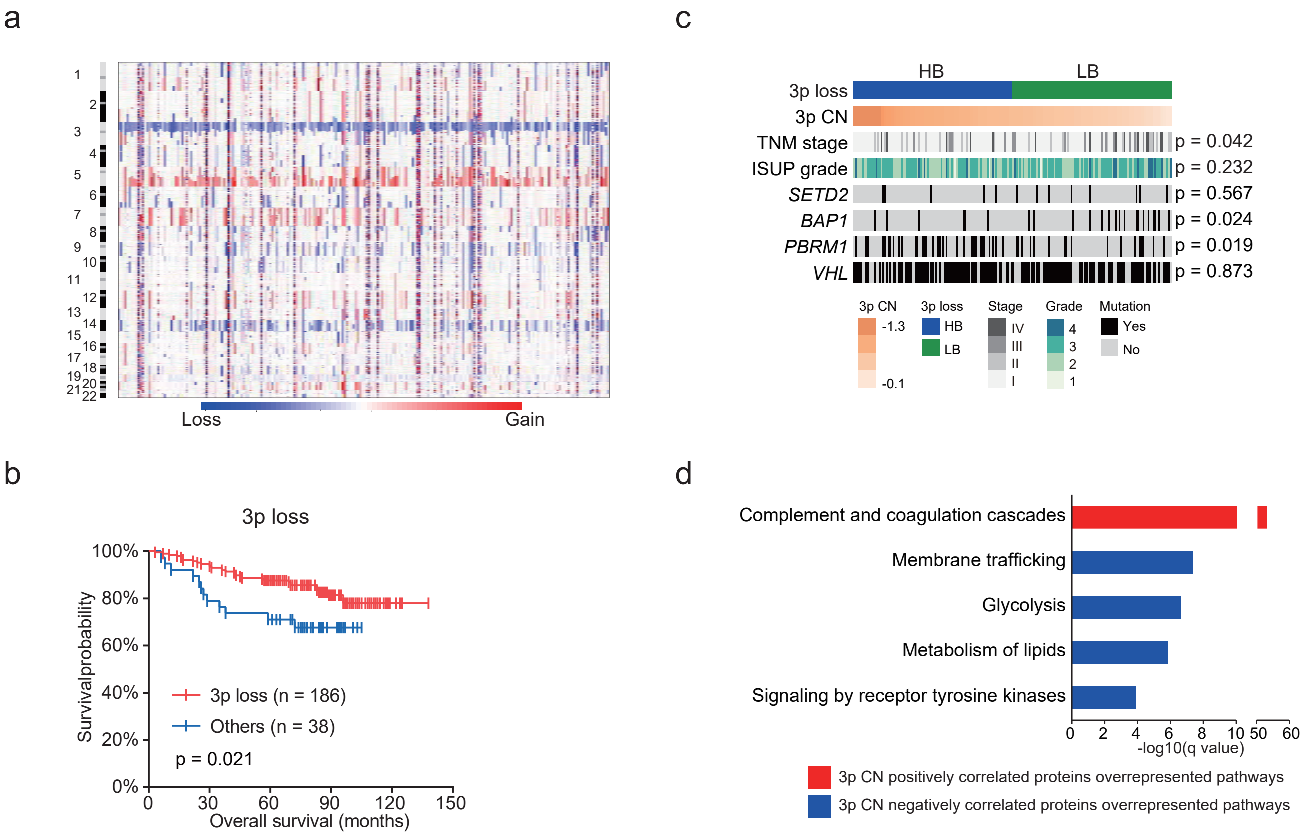

Supplementary Fig. 2 Profiles of CNAs and Effects of CNA on ccRCC Proteome.

a, CNA profiles of the Chinese ccRCC cohort. **b**, Kaplan–Meier curves of OS for patients with or without 3p loss (two-sided log-rank test). **c**, The association of 3p loss burden with clinical signatures and diver mutations in ccRCC. **d**, Chromosome 3p CN positively/negatively correlated proteins overrepresented pathways.

Supplementary Figure 3

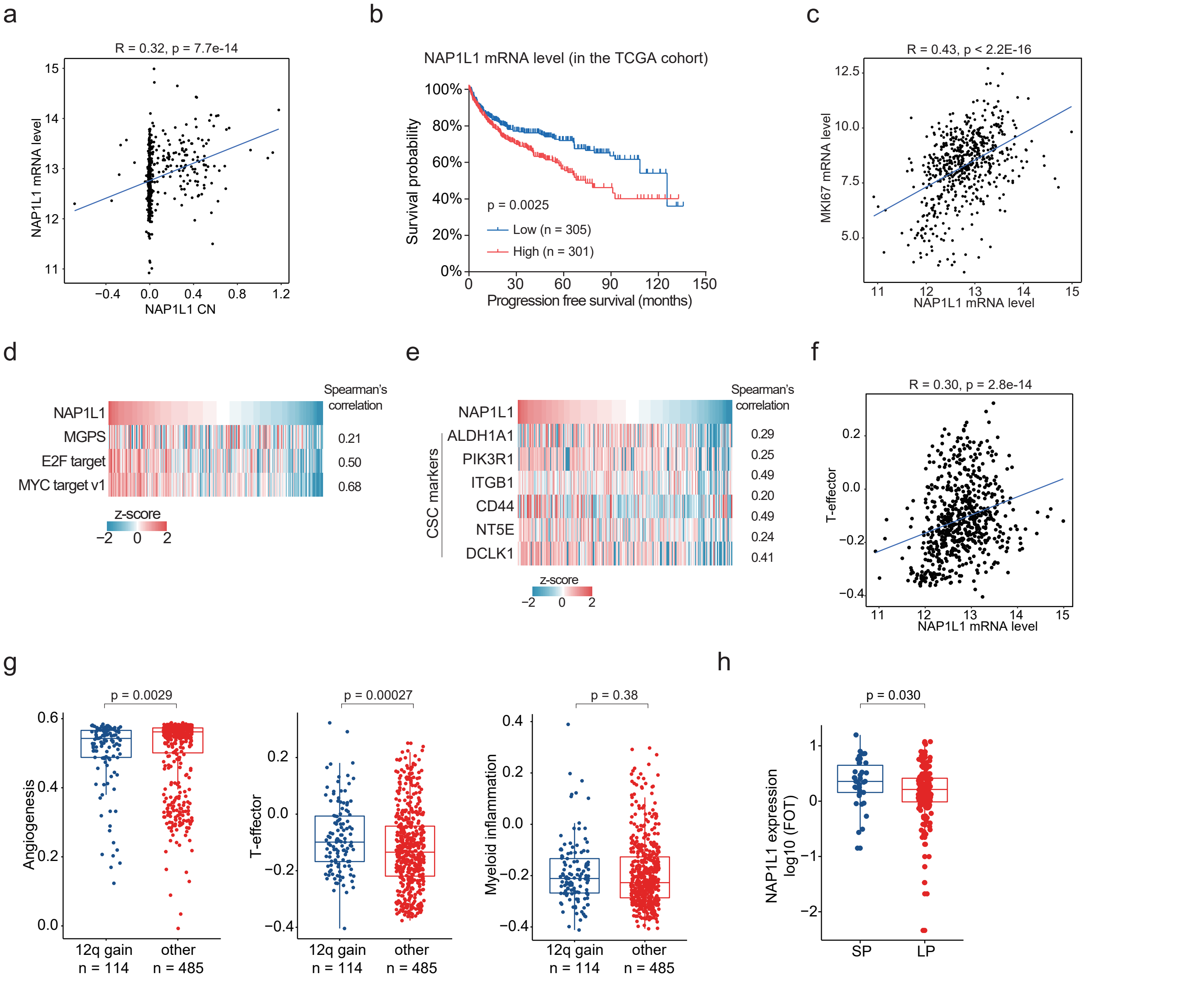

Supplementary Fig. 3 Impact of 12q Gain on Molecular and Clinical Outcomes of ccRCC.
a, The correlation between NAP1L1 CN and NAP1L1 mRNA level (two-sided Spearman's correlation test). **b**, Kaplan–Meier curves of PFS for patients with different NAP1L1 mRNA levels in the TCGA cohort. **c**, The correlation between NAP1L1 mRNA level and MKI67 mRNA level (two-sided Spearman's correlation test). **d**, The heatmap showing the correlations between NAP1L1 abundances and proliferation related pathway scores. **e**, The heatmap showing the correlations between NAP1L1 abundances and CSC marker abundances. **f**, The correlation between NAP1L1 mRNA level and T-effector score in the TCGA cohort (two-sided Spearman's correlation test). **g**, Comparison of Angiogenesis, T-effector, and Myeloid inflammation scores between ccRCC tumors with and without 12q gain in the TCGA cohort. **h**, Comparison of NAP1L1 abundances between SP and LP groups. P values are derived from two-sided t test. Boxplots in panels **g**, **h** show the median (central line), the 25–75% IQR (box limits), the ±1.5×IQR (whiskers).

Supplementary Figure 4

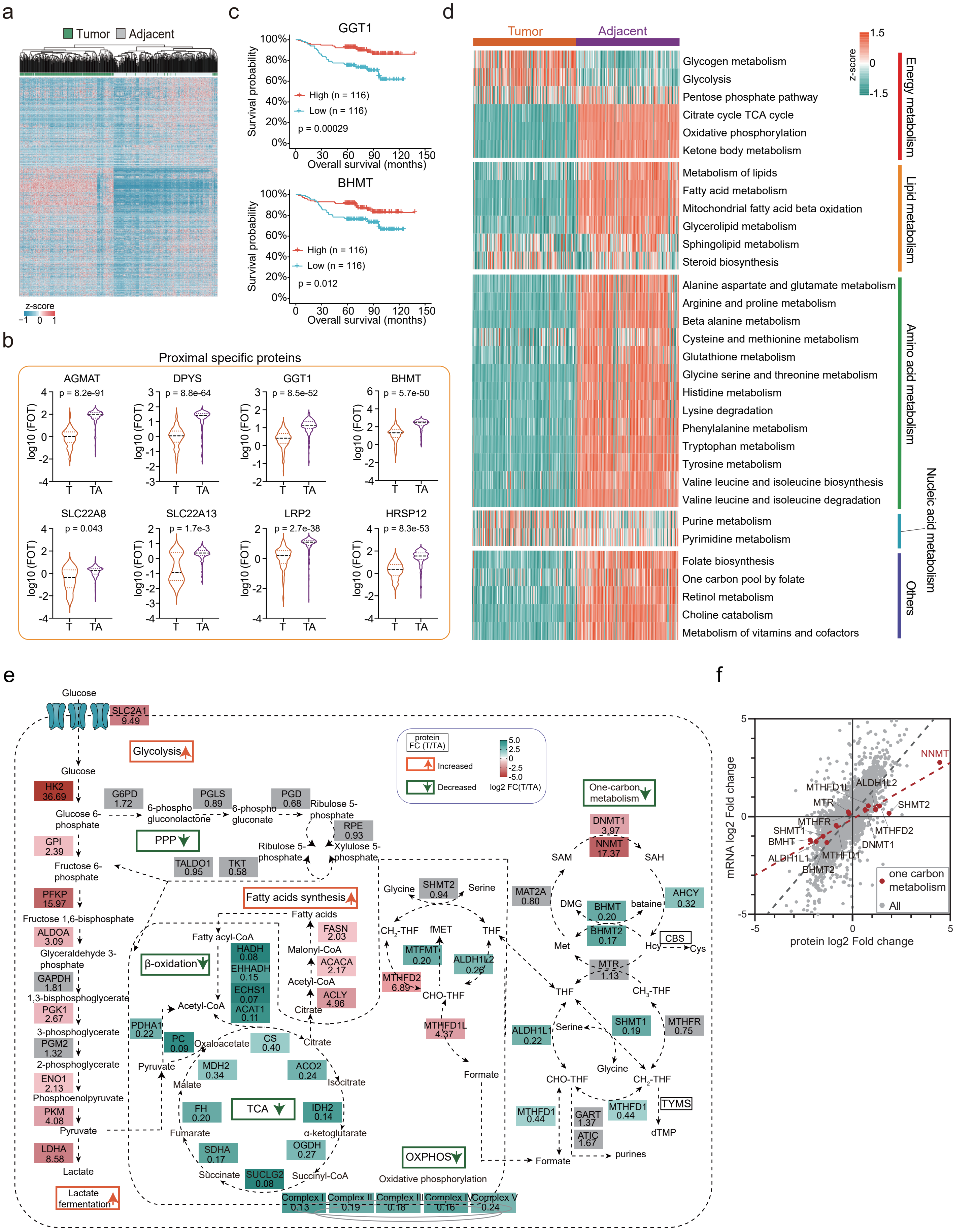

Supplementary Fig. 4 Differential Proteomic Profiles between ccRCC Tumor and Adjacent Tissues. **a**, Hierarchical clustering analysis demonstrating a clear distinction between the proteomes of tumors and tumor adjacent tissues. **b**, The eight proximal tubule specific expressed proteins annotated in Human Protein Atlas. P values are derived from two-sided t test. **c**, Two significantly downregulated renal proximal tubule signature proteins (GGT1, BHMT,) in ccRCC tumors were associated with poor prognosis (two-sided log-rank test). **d**, Metabolism-related pathway scores inferred by ssGSEA. **e**, Diagram showing broad metabolic dysregulation in ccRCC. The color gradient indicates log2 FC (T/TA) of each protein. Proteins undetected are indicated with gray boxes. **f**, Scatterplots depicting expression of protein (x axis) and mRNA (y axis). Linear regression of all mRNA-protein pairs (gray dotted line) and one-carbon metabolism mRNA- protein pairs (red dotted line) are shown.

Supplementary Figure 5

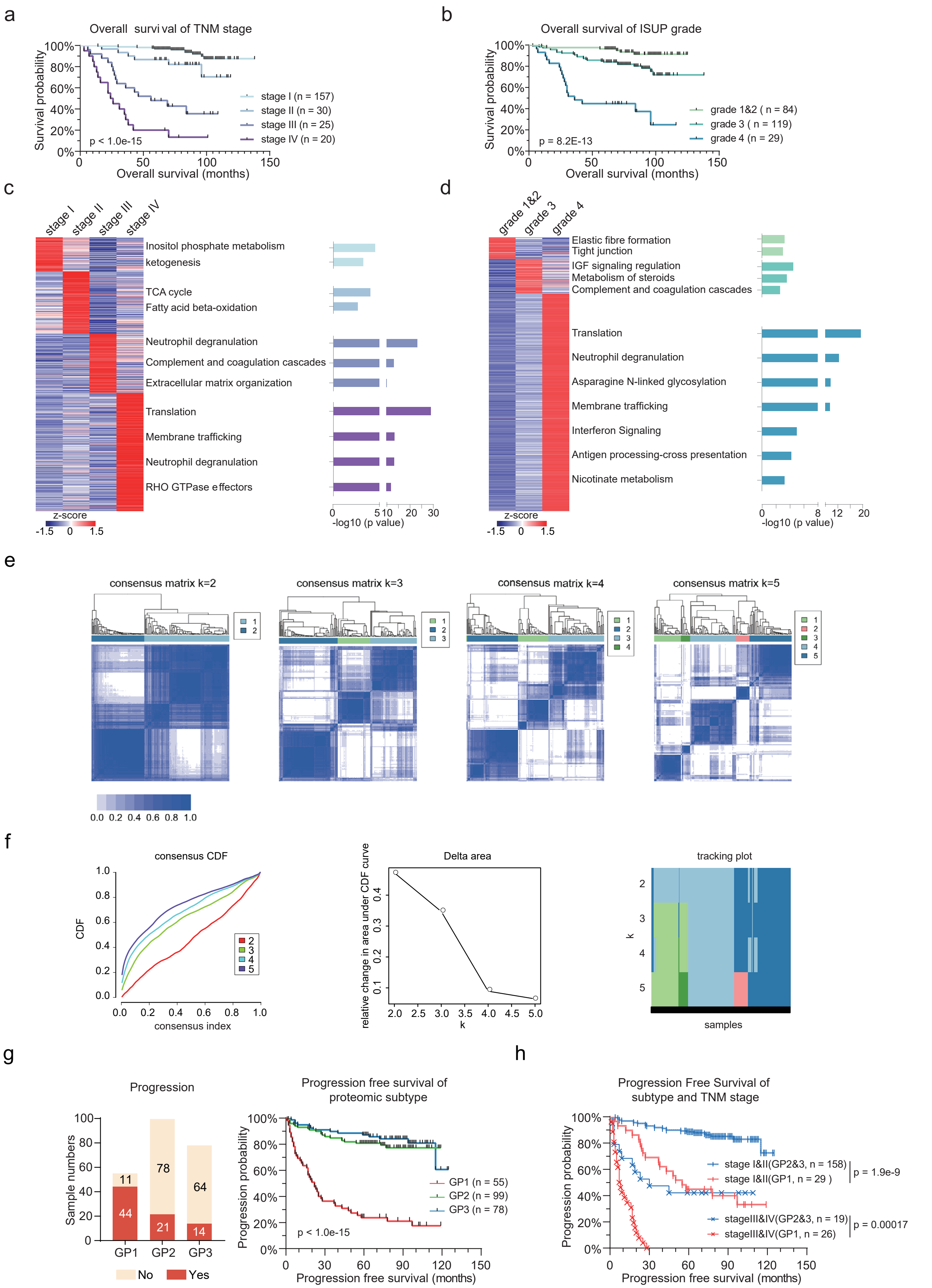

Supplementary Fig. 5 Proteomic Inter-Tumoral Heterogeneity and Consensus Clustering of ccRCC. **a**, Kaplan–Meier curves of OS for TNM stages (stage I, n = 157; stage II, n = 30; stage III, n = 25; stage IV, n = 20; two-sided log-rank test). **b**, Kaplan–Meier curves of OS for ISUP grades (grade 1, n = 3; grade 2, n = 81; grade 3, n = 119; grade 4, n = 29; two-sided log-rank test) **c**, Overrepresented pathways according to TNM stages. **d**, Overrepresented pathways according to ISUP grades. **e**, Consensus matrices of the 232 ccRCC samples from k = 2 to k = 5. Consensus clustering was conducted for the top 1,000 most-variant proteins. **f**, Cumulative distribution function plot, delta plot, and tracking plot corresponding to consensus matrices from k = 2 to k = 5. **g**, Progression status among three subtypes and Kaplan–Meier curves of PFS for the three subtypes (two-sided log-rank test). **h**, Kaplan–Meier curves of PFS for subtypes GP1 and GP2&3 at different TNM stages (stage I&II vs. III&IV) (two-sided log-rank test).

.

Supplementary Figure 6

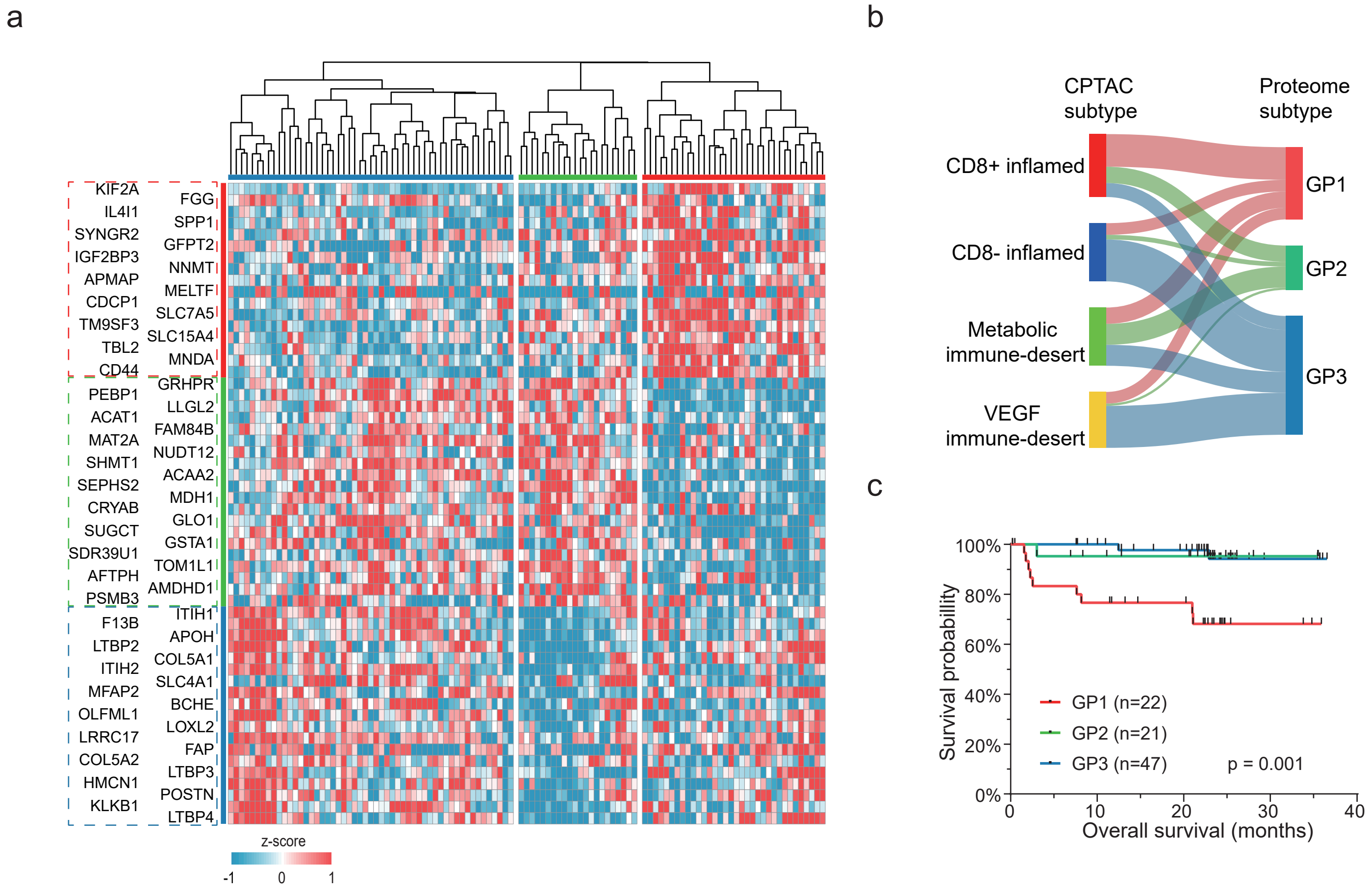

Supplementary Fig. 6 Validation of the Performance of Proteomic Subtypes.

a, Clustering of CPTAC proteomic data by the most representative proteins derived from Chinese ccRCC subtypes. **b**, Chinese proteomic subtype of the CPTAC ccRCC cohort. **c**, Kaplan–Meier curves of OS for CPTAC-GP1, CPTAC-GP2 and CPTAC-GP3 (two-sided log-rank test).

Supplementary Figure 7

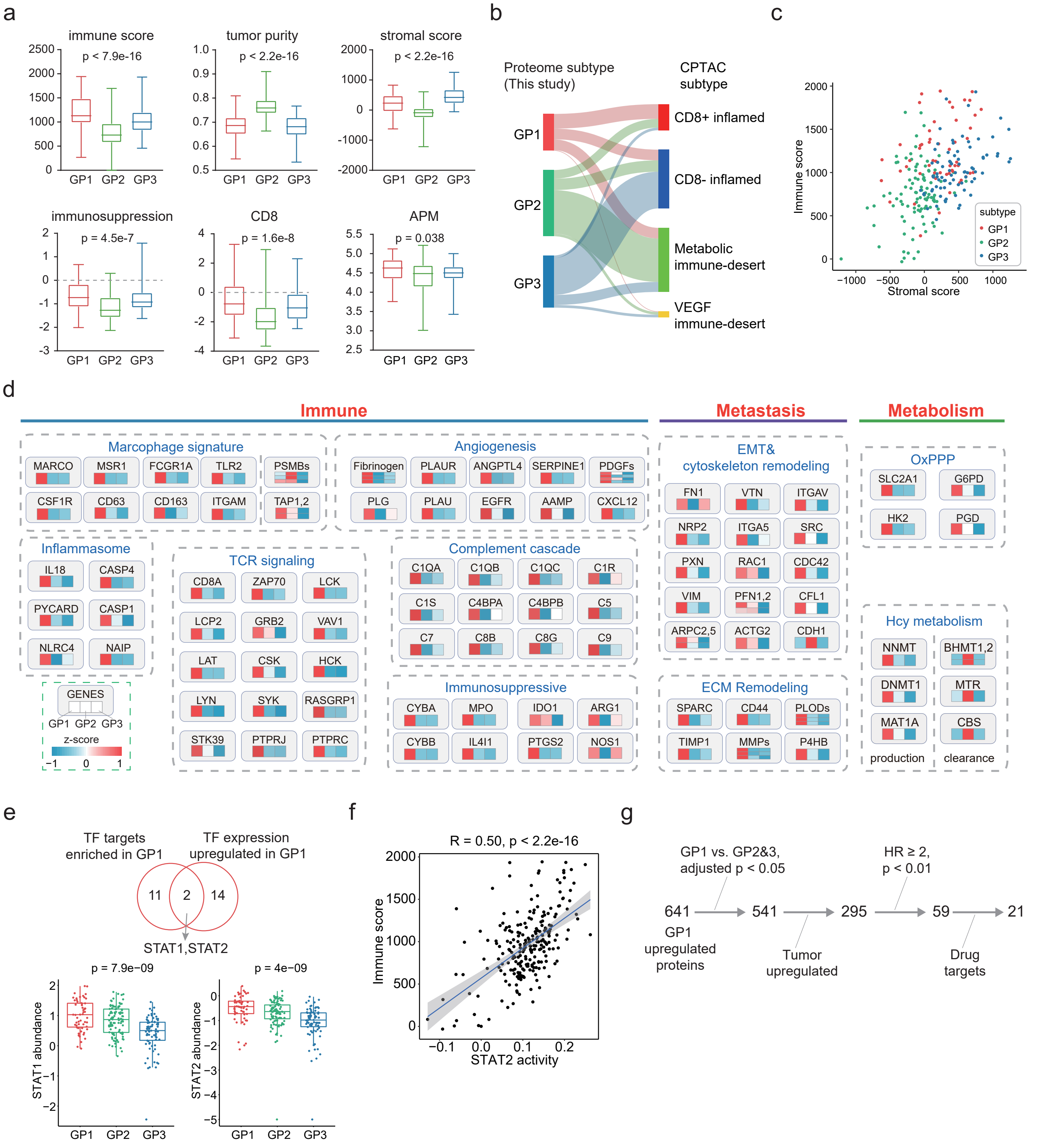

Supplementary Fig. 7 Proteomic Features of the Three ccRCC Subtypes.

a, Immune scores, stromal scores, tumor purities, and APM, immunosuppression, and CD8 cluster scores of three proteomic subtypes (GP1, n = 55; GP2, n = 99, GP3, n =78). P values are derived from Kruskal–Wallis test. Boxplots show the median (central line), the 25–75% IQR, the min–max (whiskers). **b**, Distribution of immune and stromal scores of three proteomic subtypes. **c**, Mapping of CPTAC subtype and Chinese proteomic subtype. **d**, Summary of signature proteins and pathways involved in tumor aggressiveness present in GP1. **e**, Comparison of protein abundances of STAT1 and STAT2 three proteomic subtypes (GP1, n = 55; GP2, n = 99, GP3, n =78). P values are derived from Kruskal–Wallis test. Boxplots show the median (central line), the 25–75% IQR (box limits), the ±1.5×IQR (whiskers). **f**, STAT1 activities were significantly correlated with immune scores (two-sided Spearman's correlation test). Shaded region indicates 95% confidence interval for the correlation. **g**, Strategy for prioritizing druggable targets in GP1 upregulated proteins.

.

Supplementary Figure 8

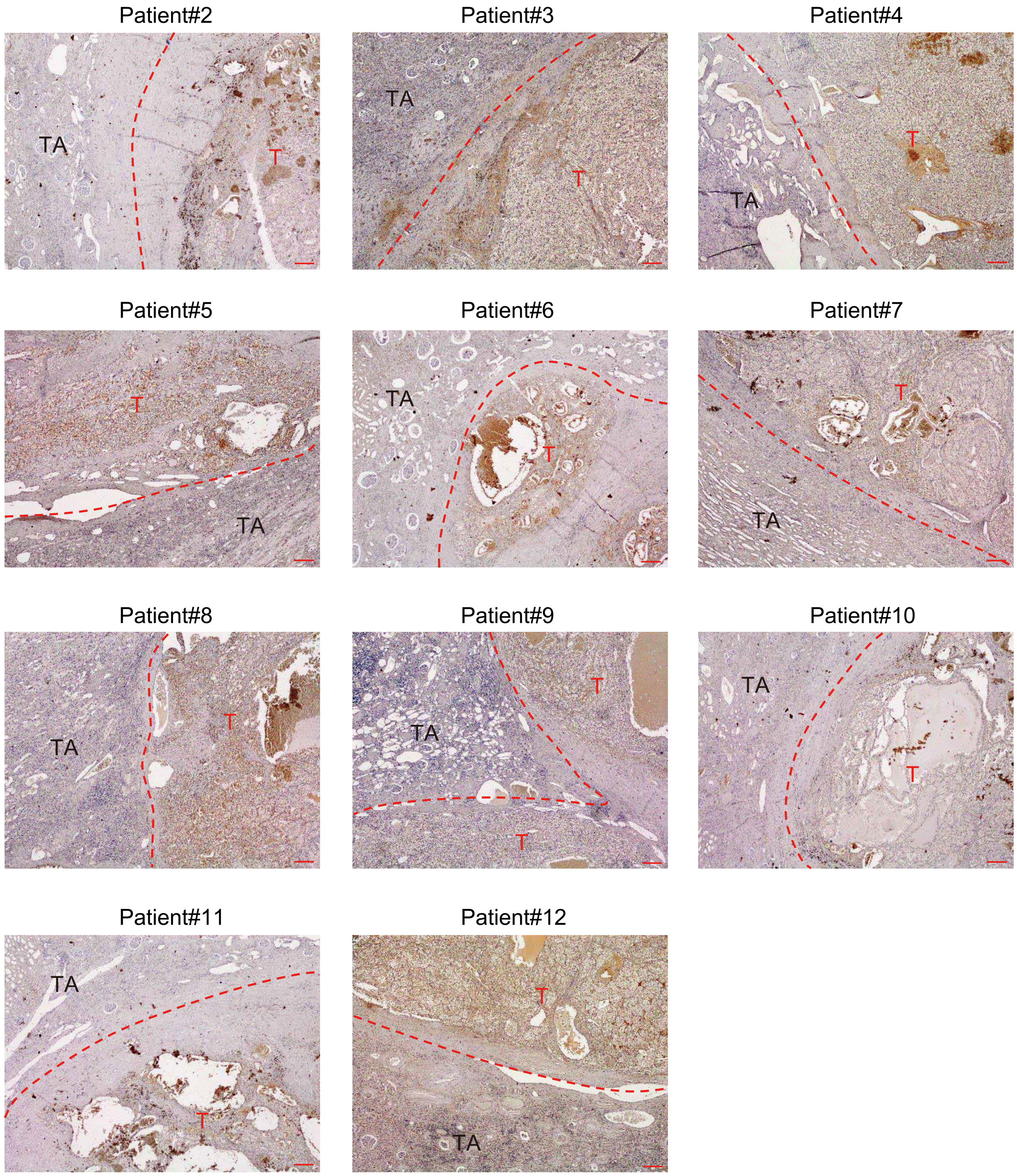

Supplementary Fig. 8 IHC Analysis for NNMT in ccRCC.

Slides of ccRCC samples were subject to IHC. Boundaries between tumor and adjacent normal tissues are marked by red dashed lines. Scale bars: 200 µm.

Supplementary Figure 9

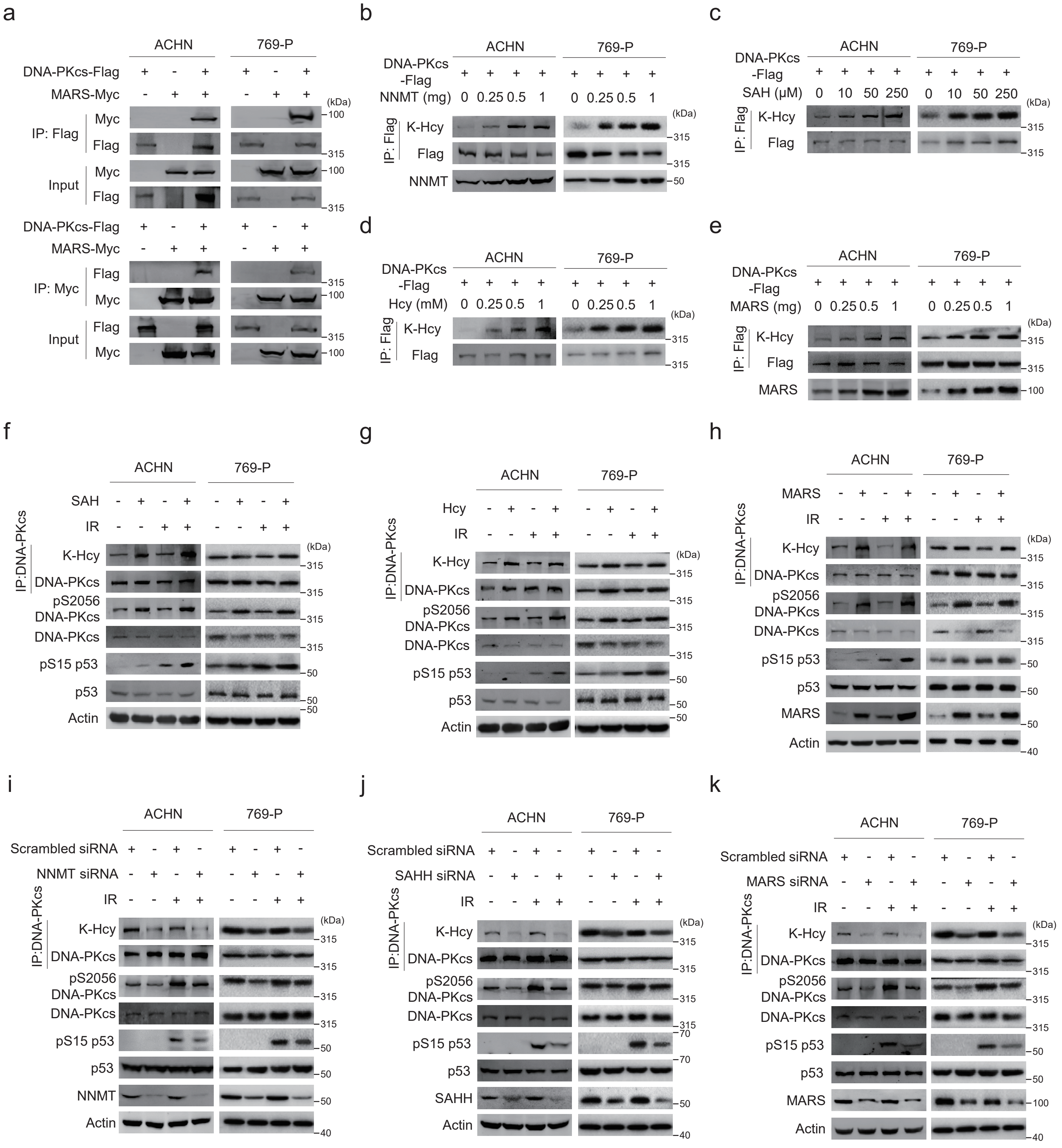

Supplementary Fig. 9 Lysine-Homocysteinylation modification of DNA-PKcs Activates Cell DNA Damage Repair.

a, Co-immunoprecipitation assay showing that exogenous DNA-PKcs and exogenous MARS interact in cultured cells (n = 3 biological repeats). **b-e**, K-Hcy levels of DNA-PKcs in cells subjected to various treatments. **f-k**, Western blot analysis of K-Hcy levels of DNA-PKcs, DNA-PKcs (pS2056), and p53 (Ser15) in cells subjected to various treatments.
