# Supplementary material for: A proteogenomic analysis of clear cell renal cell carcinoma in a Chinese population
Source: Nat Commun. 2022 Apr 19;13:2052. doi: 10.1038/s41467-022-29577-x (PMC9019091; doi:10.1038/s41467-022-29577-x)
Supplement: Supplementary file 3 — Description of Additional Supplementary Files [file 41467_2022_29577_MOESM3_ESM.pdf]

Description of Additional Supplementary Files

File Name: Supplementary Data 1
Description: Clinical characteristics of 232 Chinese ccRCC patients, survival analysis and abbreviations used in the text.

File Name: Supplementary Data 2
Description: Genomic alterations and their proteomic and clinical consequences in ccRCC.

File Name: Supplementary Data 3
Description: Proteomic alterations between ccRCC tumor and tumor adjacent tissues.

File Name: Supplementary Data 4
Description: Proteomic differences based on ISUP grade and TNM stage.

File Name: Supplementary Data 5
Description: Molecular features of proteomic subtypes, including genetic alterations, differential expressed proteins, and enriched pathways.
